# The impact of New Families home visiting program on first-time mothers’ quality of life and its association with social support: a non-randomized controlled study

**DOI:** 10.1186/s12889-023-17285-0

**Published:** 2023-12-08

**Authors:** Malene Brekke, Milada Cvancarova Småstuen, Kari Glavin, Amin Amro, Beate Solberg, Anne-Martha Utne Øygarden, Kristin Marie Sæther, Trude Haugland

**Affiliations:** 1 Centre of Diaconia and Professional Practice, VID Specialized University, Oslo, Norway; 2 Faculty of Health Sciences, VID Specialized University, Postboks 184 Vinderen, Oslo, 0319 Norway; 3 Oslo Metropolitan University, Oslo, Norway

**Keywords:** Child Health Services, First-time mother, Home visit, Pregnancy, Postpartum Period, Quality of Life

## Abstract

**Background:**

The transition to motherhood is characterized by physical, psychological, social, and relational changes. Quality of life (QoL) changes substantially during this transition. Higher QoL is associated with social support, essential for coping with the challenges and changes of becoming a mother. An early universal home visiting program (New Families) is developed to strengthen and support families. The study aims to evaluate the impact of New Families on first-time mothers’ QoL and to investigate the association between their QoL, social support, and selected possible predictive factors.

**Methods:**

A prospective non-randomized controlled study with parallel group design. Child Health Services in five city districts of Oslo were matched in intervention and control groups. First-time mothers were allocated based on the residential area and assessed at pregnancy week 28 (*N* = 228), six weeks postpartum (*N* = 184), and three months postpartum (*N* = 167). Measures of the World Health Organization Quality of Life brief, Perinatal Infant Care Social Support Scale, and background variables were collected from October 2018 to June 2020. Multivariate linear regression models were applied to examine intervention impact and assess associations.

**Results:**

Our data did not reveal a significant association between New Families intervention and the QoL levels of first-time mothers at three months postpartum. Thus, we analyzed the whole sample together. Emotional support was significantly associated with higher QoL levels in the physical health (B = 0.19, 95%CI [0.04 to 0.34]) and social relationships (B = 0.40, 95%CI [0.20 to 0.60]) domains. Appraisal support was significantly associated with higher QoL levels in the psychological (B = 0.34, 95%CI [0.18 to 0.50]) and environment (B = 0.33, 95%CI [0.19 to 0.48]) domains. QoL levels in pregnancy were significantly associated with QoL levels postpartum, showing small to medium effect size (ES = 0.30 to 0.55), depending on the domain.

**Conclusions:**

Further research, including qualitative interviews, could provide more insights into the impact of New Families on QoL. A positive association between QoL levels in pregnancy and postpartum suggests that postnatal interventions targeting improved QoL could potentially improve postpartum QoL. Emotional and appraisal support seems beneficial for first-time mothers’ QoL and could be provided and facilitated by public health nurses.

**Trial registration:**

clinicaltrial.gov *NCT04162626.*

**Supplementary Information:**

The online version contains supplementary material available at 10.1186/s12889-023-17285-0.

## Background

Transition to motherhood is characterized by physical, psychological, and social changes [1] and the development of a relationship with the newborn [2]. During this transition, women’s Quality of Life (QoL) has been shown to change substantially [3–5]. QoL can be understood as “… individuals’ perceptions of their position in life in the context of the culture and value systems in which they live and in relation to their goals, expectations, standards, and concerns” [6]. In pregnant and postpartum women, QoL related to health, encompassing physical, psychological, and social domains, is more or less affected [7, 8]. QoL attributed to physical health decreases during pregnancy before it increases in the first 12 postpartum weeks [3–5]. The psychological domain diminishes [5] or remains stable [4] during the transition, and the social domain diminishes into the postpartum period [3, 4].

In postpartum women, various aspects of support, such as adequate antenatal care consultations [9], postnatal home visits [10], relationship satisfaction [11], and support from partners, family, and friends [12, 13], are associated with higher QoL. Social support relates to social structures and functions conceptualized as informational support, which provides advice and suggestions; instrumental support, including hands-on assistance; emotional support, involving acts of empathy and care; and appraisal support, enabling self-evaluation and feedback [14]. Few studies have explored these functions of social support and its association with QoL domains in pregnant and postpartum women. During the transition to motherhood, social support is found to be a predictor of good mental health [15–18] and is associated with feelings of security [19], as well as important to increase coping and mastery of the parental role [20–22]. First-time mothers have identified a need for social support from informal and formal sources [21], which seems particularly important in first-time mothers [19].

Home visits, as formal professional support, are recommended in the WHOs strategy on maternal care [23]. Home visits are by Public Health Nurses (PHN), first-time mothers, and fathers considered a successful method for developing a relationship in a safe environment and providing support [24–27]. Nevertheless, it promotes self-confidence in the parental role [25] and may improve women’s satisfaction with postnatal care [28]. However, the effect of additional home visits on maternal health is indistinctive [28].

The Norwegian Child Health Services (CHS) are provided through the Primary Health Care Services, aiming to prevent diseases, maintain good health status for all children, and improve parents’ autonomy and independence. The CHS is the only health promotive and preventive service to children 0–5 years old at the municipal level. The CHS is free, used by 98% of the eligible population [29], and offers a child health program including consultations provided at age-specific time points [30]. Each consultation is regulated by legislation and national guidelines recommending specific time allocations and topics to be discussed, monitored and documented. All within a timeframe of approximately 30 min. At the same time, the family’s needs should be the primary focus of each consultation [30].

An early universal home visiting program, New Families (NF), has been developed by a department in the City of Oslo in close collaboration with researchers, PHNs, and users of the CHS. Further information on this development is described elsewhere [26, 31]. NF is an integrated addition to the child health program provided by the CHS [26]. NF is based on repeated home visits from pregnancy week 28 until the child is two years old and is offered to all parents (including single parents) expecting their first child. Compared to the traditional CHS, NF adds an earlier approach to the families, provides more resources for contact outside the age-specific time points, and thus, has the potential to provide a more targeted service based on each family’s needs [26, 31]. PHNs have highlighted the opportunity provided through NF to build trust and a good relationship with the parents before the child is born [31, 32]. The CHS and NF aims to strengthen and support families in the transition to parenthood and bolster the parents’ ability to cope and their confidence in the parental role [30, 33]. This salutogenic goal and its theory are particularly emphasized in NF and are crucial to its novelty [31].

The transition to motherhood is argued to be a vulnerable period, affecting aspects of QoL, and emphasizing the need for informal and formal support. The potential for PHNs to provide more formal support through NF, compared to the traditional CHS, may affect women’s QoL during the transition to motherhood. Therefore, the aims of this study were to 1) evaluate the impact of NF on first-time mothers’ QoL by exploring differences between the first-time mothers receiving NF and the first-time mothers receiving follow-up as usual, and if no impact of NF on QoL 2) investigate the association between QoL, social support and selected possible predictive factors in the sample of first-time mothers.

## Methods

### Study design

This is a prospective non-randomized controlled study with parallel group design, using data from the New Families research project, which evaluates the New Families home visiting program registered at clinicaltrials.gov (NCT04162626). The Regional Committees for medical and health research ethics in Norway (reference no: 2018/1378) and the Norwegian Centre for Research Data (project no: 735207) has approved the New Families research project. This study reports on the additional outcome QoL, which were collected in the study described above.

### Participants and setting

A department in the City of Oslo selected and matched five city districts of Oslo as either intervention or control districts. The aim of matching the groups was to achieve equality between the intervention and control districts by using data and statistics on population level regarding sociocultural factors, population composition, birth statistics, immigrant proportion, and work participation. Randomization of districts or respondents was impossible, as NF was already implemented in the CHS in several districts. Hence, this limited the number of city districts available for inclusion, and the number of variables we were able to match for had to be adjusted for accordingly. The residential district determined the respondents’ group allocation. First-time mothers and their families in the control districts received service as usual, and the intervention districts received NF in addition to service as usual.

Midwives or clinic secretaries at the CHS clinics in the five city districts recruited pregnant women attending pregnancy check-ups by screening them according to eligibility criteria and inviting them to participate. Inclusion criteria were pregnant women expecting their first child and living in one of the five city districts. The exclusion criteria were multiparous women. Women with specific conditions or diseases were not actively excluded, and no exclusion criteria were applied later based on birth outcomes. The recruiters at the CHS clinics were instructed to provide the pregnant women with initial information on the study, including a short information letter available in ten languages (Norwegian, English, Arabic, Lithuanian, Pashto, Polish, Somali, Tamil, Turkish, and Urdu). Contact information for these pregnant women was provided by the CHS clinics to researchers in the NF research project who contacted the pregnant women for inclusion in the study. The current study only includes pregnant women/first-time mothers. However, in the NF research project the women’s partners were recruited to the study by the researchers when contacting the pregnant women interested in participating.

The recruitment was conducted from October 2018 to December 2019. Of the 427 pregnant women invited to participate, we included 228 pregnant women, divided by 124 pregnant women in the intervention districts and 86 pregnant women in the control districts. Due to General Data Protection Regulation laws, we were not allowed to collect any information on non-respondents.

Power calculation for the NF research project were estimated based on the outcome measure of Edinburgh Postnatal Depression Scale [34]. The calculation was based on a power of 0.80, an alpha level of 0.05, and an effect size of 0.5 [35, 36]. It was calculated that we needed 64 participants in each group. Hence, the sample size of this study was determined by the number of respondents eligible and willing to participate in the study.

### Data collection

All pregnant women interested in participating received informed consent, by mail, for written completion. Self-reported measures were sent to first-time mothers by mail from October 2018 to June 2020. Data were collected at three time points: around pregnancy week 28 (T1), six weeks postpartum (T2), and three months postpartum (T3). The consent form and all measures were available in ten languages.

### Control

**Fig. 1:**
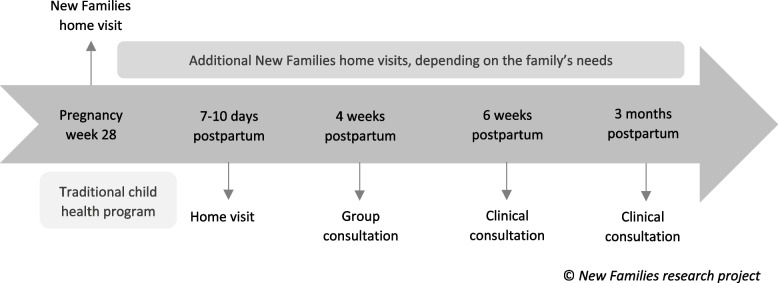
Timeline of the New Families home visiting program in the context of the traditional child health program

The pregnant women/first-time mothers in the control districts received the traditional child health program provided by the CHS. Up to three months postpartum, the CHS provides one home visit 7–10 days postpartum, one group consultation, and two clinical consultations [30]. These consultations include information, guidance, and monitoring related to physical, psychological, social, and sexual health and development, and the parents’ health. The group consultation provides information on specific health and development topics and is conducted together with other parents with babies around the same age. No consultations with the PHN are offered during pregnancy. The timeline of the NF home visiting program in the context of the traditional child health program is presented in Fig. 1.

### Intervention

The pregnant women/first-time mothers in the intervention districts received NF in addition to the traditional child health program offered by the CHS, as described under *Control*. The first NF home visit was offered around pregnancy week 28. The home visit was conducted by a PHN and scheduled for 1 to 1.5 hours. Further, the number and frequency of home visits depended on the family’s needs. The intention was that the same PHN should follow up the families across all consultations and home visits provided by NF and at the CHS clinics. This is not a prerequisite in the CHS. NF is an unstandardized intervention as it is tailored to the unique needs of each family [33].

NF is based on a salutogenic perspective, focusing on resource mobilization, using methods of motivational interviewing and empathic communication. NF is regulated by a manual [33], serving as a governing document. It includes theoretical and practical information related to the conduct of NF. The PHNs received theoretical training on the NF manual related to the methods, theory, and background of NF, and practical training and guidance from experienced PHNs in the first home visit during pregnancy. The PHNs wrote reflection notes from the home visits, a tool for reflecting on their practice, and used as a documentation on the theory and method underpinning NF [33].

### Measures

#### Demographics and selected possible predictive factors

Standard demographic data were measured at T1 and included family income, educational level, age, nationality, and marital status. Single items at T1 assessed the presence of complications during pregnancy, previous and present mental health conditions, hours of sleep, and the perception of enough/not enough sleep (also assessed at T2).

#### Quality of life

QoL is measured at T1 and T3 by the World Health Organization Quality of Life Questionnaire brief version (WHOQOL-BREF) [37], containing 26 items. Two single items on overall QoL and general health satisfaction are examined separately but are not reported in this study. The remaining 24 items construct the domains physical health (7 items), psychological (6 items), social relationship (3 items), and environment (8 items). All items are assessed on a 5-point Likert scale. Higher values indicate higher QoL. The domain scores, ranging from 4–20, were calculated by multiplying the mean score of each domain by four, according to the WHOQOL-BREF scoring manual [37]. The Norwegian WHOQOL-BREF version is valid and reliable [38, 39], and the instrument is reported to be valid and reliable in pregnant and postpartum women [40, 41]. In the present study, Cronbach’s alpha at T1/T3 was 0.80/0.71, 0.81/0.83, 0.67/0.70, and 0.78/0.78 for the physical health, psychological, social relationship, and environment domains, respectively.

#### Social support

Social support is measured at T2 by *Perinatal Infant Care Social Support* (PICSS) [14], containing two scales. The PICSS Functional scale assesses the women’s perception of 22 statements which constructs the four domains: informational (7 statements), instrumental (7 statements), emotional (4 statements), and appraisal (4 statements) support. Each statement is assessed on a 4-point Likert scale, scored from 1–4. Higher scores indicate higher social support. Each domain is scored by the sum score of all statements included. The PICSS Structural scale identifies persons that provide at least one type of functional support. The persons were grouped into formal (PHN, midwife, general practitioner, health care professionals – maximum of four persons) and informal (partner, parents, parents-in-law, siblings, friends, neighbors – maximum of nine persons) sources and scored by counting the number of persons identified as respectively formal and informal sources of support. The instrument is developed and validated for use in postpartum women in Ireland [14, 20]. PICSS was translated and re-translated to Norwegian for this research project. In our study, Cronbach’s alpha was 87, 0.83, 0.85, and 0.81 for the informational, instrumental, emotional, and appraisal domains.

### Statistical methods

Descriptive statistics were used to describe the sample characteristics. Continuous variables were described by means and standard deviations (SD) and categorical data as counts and percentages. Crude comparisons between pairs of variables were performed using t-test for continuous variables and chi-square for categorical variables.

As the dependent variable (QoL) was assessed at two time points, all models were constructed with QoL variable assessed at T3 and adjusted for QoL variable measured at T1 to account for the design and possible statistical dependencies as the same individuals were assessed two times. All models are conducted with listwise exclusion, and the number of participants used in each model is marked in Tables 2 and 3. We did not impute missing values as our sample did not have sufficient statistical power to perform model based imputation and the proportion of missing values was limited. Model fit for all the presented models was checked using visual inspection of residual plots and all residuals followed standard normal distribution.

A linear regression model was fitted with QoL as the dependent and intervention vs. control groups (labeled: Intervention) as the independent variable. We constructed one regression model for each of the four QoL domains at T3. All the models were adjusted for baseline differences between the intervention and control groups. Further, we constructed an interaction term to test whether the effect of the intervention on our outcome depended on the QoL level at T1, e.g., that the intervention had a larger effect on those who had lower QoL in pregnancy. This variable is labeled Intervention*QoL domain and included as an independent variable.

As no differences in the outcome were found between the intervention and control groups, a two-step linear regression with a backward selection method was used to investigate the association between QoL domains, social support, and the selected possible predictive factors in the whole sample of first-time mothers. We tested four regression models, one for each of the four QoL domains assessed at T3. In the first step of each model, we entered the four subscales of the PICSS functional scale (informational, instrumental, emotional, appraisal) and PICSS structural scale (formal and informal supporters) assessed at T2, and the QoL domain assessed at T1. In the second step, we retained the variables that were statistically significant from step one and included the selected possible predictive factors (family income, pregnancy week at T1, age of the mother, and perception of sleep at T1 and T2). The criterion for removing variables from the model in the linear regression with backward selection method was set to *p*-value > 0.1.

The results of all linear regressions are presented as unstandardized regression coefficient (B), with 95% confidence interval for B (95% CI), and *p*-value. In addition, to compare the impact of each of the included covariates, we calculated effect size (ES) using Cohens d, reported as the standardized beta (β), and interpreted as small > 0.2, medium > 0.5, and large > 0.8 [42].

Sensitivity analyses were conducted to explore if answering the survey after the outbreak of COVID-19, which was on March 12^th^, 2020, in Norway, affected our analyses. We replicated the abovementioned linear regression analyses without respondents answering after the outbreak of COVID-19 at T3 and compared them to the analyses conducted on all respondents.

Internal consistency reliability was examined by calculating Cronbach’s alpha for all WHOQOL-BREF domain scales and subscales of the PICSS functional scale.

All statistical analyses were conducted in SPSS, version 28, in the secure platform of Services for Sensitive Data [43]. The level of statistical significance was set to p < 0.05 for all analyses, and all point estimates are reported with 95% CI.

## Results

**Fig. 2:**
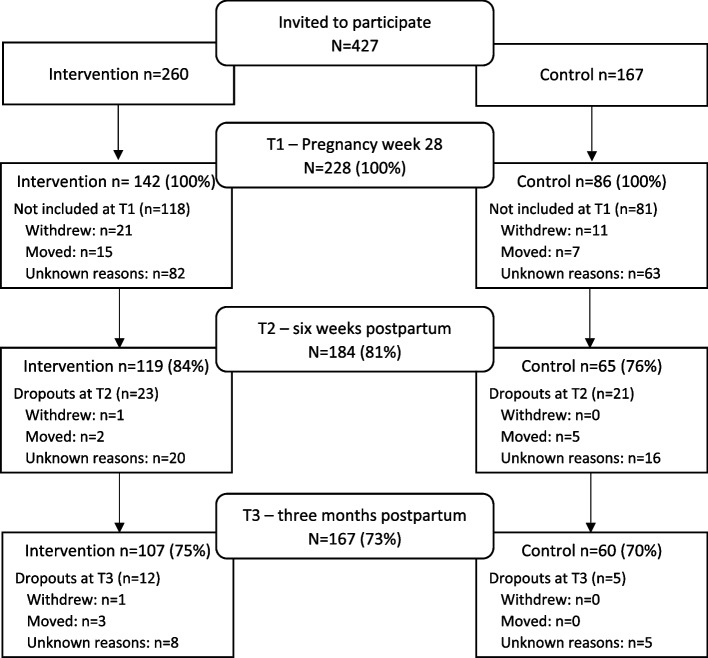
Flow chart of first-time mothers at T1, T2, and T3, with reasons for dropouts

Of the 427 first-time mothers invited to participate in the study, 228 (53.4%) were included at T1. The number of participating first-time mothers, response rates at each time point, and the dropouts by intervention and control group are presented in the flow chart in Fig. 2.

First-time mothers completed the measures in three languages. Norwegian was used by 95.6% (*n* = 218), 96.7% (*n* = 178) and 97.6% (*n* = 163) at T1, T2 and T3, respectively. The remaining respondents used English and Arabic languages (in descending order).

In the intervention group, non-respondents (*n* = 35) and respondents (*n* = 107) at T3 in did not differ statistically significantly on the background variables age (*p* = 0.178), educational level (*p* = 0.325) and family income (*p* = 0.578). In the control group, non-respondents (*n* = 26) at T3 were statistically significantly younger (*p* = 0.019) compared to the respondents (*n* = 60), and they did not differ on educational level (*p* = 0.085) or family income (*p* = 0.657).

### Sample characteristics

Most of the first-time mothers in both groups were between 29–35 years, partnered or married, had higher levels of family income, achieved a high educational level, and were Norwegians (Table 1). The intervention and control groups were similar concerning background variables at T1, except for higher levels of family income and higher pregnancy week at T1 in the intervention group and a higher percentage reporting the perception of enough sleep in the control group.

**Table 1:** Sample characteristics of first-time mothers in the control (*n* = 86) and intervention (*n* = 142) groups at T1, with crude comparisons

	**Control** **(*N* = 86)**				**Intervention** **(*N* = 142)**				**Comparison of groups**
	**N** (missing)	**%**	**Mean** (min–max)	**SD**	**N** (missing)	**%**	**Mean** (min–max)	**SD**	***p*-value**
**Age**	86 (0)	100	30.99 (22–42)	3.93	142 (0)	100	31.62 (22–47)	4.0	.25
< 28	20	23.2			26	18.3			
29–35	54	62.8			95	66.9			
36 <	12	14			21	14.8			
**Pregnancy week (at T1)**	85 (1)	98.8	30.20 (26–39)	2.79	137 (5)	96.5	32.68 (27–40)	3.73	** < .001**
Week 26–30	55	64.0			49	34.5			
Week 31–35	26	30.2			48	33.8			
Week 36–41	4	4.6			40	28.2			
**Marital status**	84 (2)	97.7			133 (9)	93.7			NA
Single	4	4.6			3	2.1			
Partnered	52	60.5			78	55.0			
Married	28	32.6			52	36.6			
**Educational level**	86 (0)	100			141 (1)	99.3			.076
Primary/secondary school	10	11.6			12	8.5			
College/university (< 4 years)	31	36.1			34	23.9			
College/university (≥ 4 years)	45	52.3			95	66.9			
**Family income, before tax (NOK)**	84 (2)	97.7			138 (4)	97.2			**.014**
< 750.000	15	17.5			21	14.8			
750.000–1.000.000	34	39.5			33	23.2			
1.000.000 <	35	40.7			84	59.2			
**Nationality**	86 (0)	100			142 (0)	100			1.0
Norway	74	86.0			122	85.9			
Other	12	14.0			20	14.1			
**Complications during pregnancy**	86 (0)	100			142 (0)	100			.36
Yes	23	26.7			30	21.1			
**Mental health condition**	86 (0)	100			141 (1)	99.3			
Present	3	3.5			6	4.2			1.0
Previous	10	11.6			20	14.1			.73
**Sleep, hours**	80 (6)	93.0	7.43 (3–10)	1.06	136 (6)	95.8	7.31 (3.5–12)	1.46	.54
**Perception of sleep**	86 (0)	100			142 (4)	97.2			**.034**
Enough	61	70.9			77	54.2			
Not enough	25	29.1			61	43.0			

*NA* Not applicable

In the intervention group, more than two thirds (*n* = 99, 69.7%) of the first-time mothers received the additional NF home visit around pregnancy week 28. Twenty-four (16.9%) first-time mothers did not receive this home visit, and 19 (13.4%) did not provide this information.

### Evaluating the impact of the New Families home visiting program

When adjusted for possible confounders, we did not find any statistically significant impact of the NF home visiting program on the four QoL domains when exploring the differences between the intervention and control group (Table 2). The possible confounders were baseline (T1) differences between the intervention and control group (pregnancy week, family income, perception of sleep) and QoL assessed at baseline and the interaction term intervention*QoL (assessed at baseline).

**Table 2:** Impact of New Families on WHOQOL-BREF domains at three months postpartum (T3) in first-time mothers (*n* = 156)

T1 variables	QoL domain T3															
	Physical health				Psychological				Social relationships				Environmental			
	B	95% CI	ES	*p*-value	B	95% CI	ES	*p*-value	B	95% CI	ES	*p*-value	B	95% CI	ES	*p*-value
Intervention (ref control)	-.47	-3.91 to 2.98	-.12	.790	-3.06	-7.59 to 1.47	-.62	.184	2.30	-2.01 to 6.62	.41	.293	3.24	-1.79 to 8.26	.80	.205
QoL domain	.25	.06 to .44	.34	.009	.61	.37 to .84	.54	< .001	.74	.53 to .95	.69	< .001	.71	.47 to .95	.65	< .001
Intervention*QoL domain	-.01	-.23 to .22	-.02	.971	.16	-.13 to .44	.52	.271	-.14	-.41 to .14	-.38	.332	-.21	-.51 to .08	-.90	.158
Pregnancy week	.08	-.01 to .17	.16	.056	.06	-.03 to .14	.09	.191	.09	-.02 to .19	.11	.096	.07	-.01 to .15	.13	.077
Family income (three levels)	.33	-.06 to .72	.13	.095	.39	-.02 to .79	.12	.060	-.09	-.57 to .39	-.02	.708	.23	-.16 to .61	.08	.249
Perception of sleep (ref not enough sleep)	.06	-.55 to .67	.02	.846	.08	-.54 to .70	.02	.801	.02	-.71 to .74	.01	.960	.08	-.46 to .63	.02	.760

Variables added to adjust for baseline (T1) differences between intervention and control group: pregnancy week, family income and perception of sleep. In addition, we adjusted for QoL domain assessed at baseline (T1) and added the interaction term intervention*QoL

*QoL* Quality of life

### Association between quality of life and social support

The models for each QoL domain and associations with social support and selective possible predictive factors are shown in Table 3. For the physical health domain, higher QoL levels was statistically significantly associated with higher emotional support levels (B = 0.19, 95% CI [0.04 to 0.34]), showing negligible effect size (ES = 0.17). First-time mothers with higher physical health QoL at T1 (B = 0.22, 95% CI [0.12 to 0.32]) and the perception of enough sleep at T2 (B = 1.30, 95% CI [0.79 to 1.80]) were both statistically significantly associated with higher QoL levels at T3, reaching small effect size. In the model of the psychological domain, higher QoL levels was statistically significantly associated with higher appraisal support levels (B = 0.34, 95% CI [0.18 to 0.50]), exhibiting a small effect size. Higher psychological QoL at T1 (B = 0.62, 95% CI [0.49 to 0.75]) was statistically significantly associated with the outcome and reached a medium effect size. In the social relationship domain model, higher QoL levels was statistically significantly associated with higher emotional support levels (B = 0.40, 95% CI [0.20 to 0.60]), showing small effect size. The social relationship QoL at T1 (B = 0.52, 95% CI [0.40 to 0.70]) was statistically significantly associated with the outcome and the only covariate adjusted for in the model that reached an effect size above 0.2, showing small to medium effect (ES = 0.48). In the model of the environment domain, higher QoL levels was statistically significantly associated with higher appraisal support levels (B = 0.33, 95% CI [0.19 to 0.48]), showing small effect size. The covariate environment QoL at T1 (B = 0.43, 95% CI [0.28 to 0.58]) exhibited a small effect on the outcome.

**Table 3:** Association between WHOQOL-BREF domains at T3, social support domains (PICSS) at T2 and selective possible predictive factors in first-time mothers (*n* = 152)

	**Step One** QoL domains and social support domains entered								**Step two** Statistically significant from step one and possible selective predictive factors added							
	**Full model**				**Final model**				**Full model**				**Final model**			
	**B**	**95% CI**	**ES**	***p*-value**	**B**	**95% CI**	**ES**	***p*-value**	**B**	**95% CI**	**ES**	***p*-value**	**B**	**95% CI**	**ES**	***p*-value**
***Physical health domain***																
PICSS Informational	.10	-.01 to .20	.18	.053												
PICSS Instrumental	-.08	-.18 to .02	-.15	.103												
PICSS Emotional	.17	-.11 to .44	.15	.241	.29	.13 to .46	.26	< .001	.18	.04 to .33	.17	.016	**.19**	**.04 to .34**	**.17**	**.016**
PICSS Appraisal	.13	-.17 to .43	.11	.382												
PICSS Informal support	.11	-.06 to .28	.10	.188												
PICSS Formal support	-.20	-.46 to .06	-.12	.124												
Physical health QoL domain, T1	.25	.14 to .36	.33	< .001	.27	.16 to .38	.36	< .001	.25	.14 to .36	.33	< .001	**.22**	**.12 to .32**	**.30**	** < .001**
Pregnancy week, T1									.06	-.01 to .13	.11	.10				
Age of mother									-.10	-.16 to -.04	-.22	.002	**-.09**	**-.16 to -.03**	**-.20**	**.003**
Perception of sleep, T1 (ref not enough sleep)									-.29	-.83 to .25	-.08	.293				
Perception of sleep, T2 (ref not enough sleep)									1.32	.81 to 1.83	.36	< .001	**1.30**	**.79 to 1.80**	**.35**	** < .001**
Family income (three levels)									.36	.01 to .70	.14	.045	**.39**	**.04 to .73**	**.15**	**.029**
***Psychological domain***																
PICSS Informational	.06	-.04 to .16	.08	0.260												
PICSS Instrumental	-.09	-.19 to .01	-.14	0.060												
PICSS Emotional	-.00	-.27 to .27	-.00	0.994												
PICSS Appraisal	.45	.15 to .75	.31	0.003	.41	.24 to .58	.29	< .001	.34	.18 to .50	.25	< .001	**.34**	**.18 to .50**	**.25**	** < .001**
PICSS Informal support	.02	-.15 to .18	.01	0.846												
PICSS Formal support	-.02	-.28 to .24	-.01	0.894												
Psychological domain QoL, T1	.61	.48 to .73	.57	< 0.001	.63	.50 to .75	.59	< .001	.62	.48 to .75	.55	< .001	**.62**	**.49 to .75**	**.55**	** < .001**
Pregnancy week, T1									.04	-.04 to .12	.06	.305				
Age of mother									.01	.06 to .08	.01	.832				
Perception of sleep, T1 (ref not enough sleep)									.12	-.45 to .70	.03	.675				
Perception of sleep, T2 (ref not enough sleep)									.65	.08 to 1.22	.14	.025	**.70**	**.14 to 1.25**	**.15**	**.014**
Family income (three levels)									.32	-.06 to .70	.10	.097	.36	-.01 to .73	.11	.056
***Social relationships***																
PICSS Informational	.07	-.06 to .19	.08	.303												
PICSS Instrumental	-.06	-.19 to .06	-.08	.320												
PICSS Emotional	.28	.06 to .63	.17	.104	.44	.23 to .65	.27	< .001	.40	.19 to .60	.25	< .001	**.40**	**.20 to .60**	**.25**	** < .001**
PICSS Appraisal	.20	-.17 to .57	.12	.282												
PICSS Informal support	.20	-.00 to .40	.12	.052	.18	-.02 to .37	.11	.077								
PICSS Formal support	-.16	-.48 to .16	-.06	.325												
Social relationship QoL domain, T1	.54	.40 to .69	.50	< .001	.57	.42 to .71	.52	< .001	.52	.39 to .66	.48	< .001	**.52**	**.40 to .70**	**.48**	** < .001**
Pregnancy week, T1									.11	.02 to .20	.14	.020	**.11**	**.02 to .20**	**.14**	**.017**
Age of mother									-.10	-.18 to -.02	-.14	.021	**-.09**	**-.18 to -.01**	**-.14**	**.022**
Perception of sleep, T1 (ref not enough sleep)									-.12	-.80 to .56	-.02	.72				
Perception of sleep, T2 (ref not enough sleep)									.76	.09 to 1.43	.14	.027	**.73**	**.08 to 1.38**	**.14**	**.028**
Family income (three levels)									.05	-.40 to .49	.01	.838				
***Environment***																
PICSS Informational	.09	-.01 to .18	.15	.064												
PICSS Instrumental	-.04	-.13 to .05	-.07	.383												
PICSS Emotional	.02	-.23 to .26	.02	.881												
PICSS Appraisal	.34	.07 to .61	.29	.013	.40	.24 to .56	.34	< .001	.33	.19 to .48	.30	< .001	**.33**	**.19 to .48**	**.30**	** < .001**
PICSS Informal support	.05	-.10 to .20	.05	.485												
PICSS Formal support	.06	-.17 to .29	.03	.634												
Environment QoL domain, T1	.43	.28 to .57	.41	< .001	.46	.32 to .60	.44	< .001	.43	.28 to .58	.39	< .001	**.43**	**.28 to .58**	**.39**	** < .001**
Pregnancy week, T1									.06	-.01 to .12	.10	.101	.06	-.01 to .12	.10	.099
Age of mother									-.08	-.14 to -.02	-.16	.013	**-.08**	**-.14 to -.02**	**-.16**	**.012**
Perception of sleep, T1 (ref not enough sleep)									.02	-.48 to .53	.01	.931				
Perception of sleep, T2 (ref not enough sleep)									.55	.05 to 1.04	.14	.030	**.55**	**.07 to 1.03**	**.14**	**.025**
Family income (three levels)									.32	-.03 to .67	.12	.075	.32	-.03 to .67	.12	.073

*QoL* Quality of life

### Sensitivity analyses

We replicated the analyses in Tables 2 and 3 without the 23 (13.8%) first-time mothers answering T3 after the outbreak of COVID-19. The sensitivity analyses confirmed the results from the main analysis of the impact of NF home visiting program on the QoL domains (Supplementary Table S1).

Regarding the analyses of the association between QoL and social support, the sensitivity analyses revealed only small changes compared to the main analyses (Supplementary Table S2). Variables that were removed or increased their impact to be statistically significant in the sensitivity analyses had the highest effect size of 0.25 and 0.22, respectively, thus the effect was small. The remaining such variables had effect size below 0.20, showing negligible effect. None of the regression coefficients changed direction in the sensitivity analyses.

## Discussion

This study aimed to evaluate NF’s impact on first-time mothers’ QoL and to investigate the association between their QoL, social support, and selected possible predictive factors. Our data did not reveal that NF impacted first-time mothers’ QoL at three months postpartum. Social support through a supporting presence at six weeks postpartum was associated with higher QoL at three months postpartum. In addition, the study showed that the QoL during the third trimester of pregnancy was related to the QoL at three months postpartum.

Our study’s null finding regarding NF’s impact on QoL may imply that the traditional program provided by the CHS is sufficient in maintaining first-time mothers’ QoL levels during the given period. Thus, until further outcomes of NF are evaluated, politicians and clinical practitioners may continue with NF. Further research, such as qualitative interviews, may provide more insight into the first-time mother’s experiences with NF regarding QoL and related factors.

The abovementioned finding could be due to several reasons. *First*, the relatively short timeframe of the study, from the third trimester of pregnancy to three months postpartum, may have made it difficult to detect a statistically significant change in QoL. The traditional program offered by the CHS already provides close follow-up [30] during this vulnerable and stressful period [1], which may have further hindered the ability to detect differences between the groups. *Second*, we did not measure PHNs fidelity to NF manual or implementation determinants related to internal and external factors of the CHS. This is important aspects when evaluating the achievement of the desired service delivery and clinical outcomes of NF [44, 45], such as increased QoL. *Third*, first-time mothers in our study may have changed their internal standards and values concerning QoL during the transition to parenthood, making the units of comparison for QoL during pregnancy irrelevant [46]. Thus, a response shift may have reduced the impact of the intervention. *Fourth*, using a period-specific (pregnancy/postpartum) instead of a generic instrument may have assessed QoL constructs that are more relevant to pregnant and postpartum women and, thus, yielded different results [47]. *Fifth*, the complexity of public health interventions and unstandardized interventions like NF makes them challenging to evaluate [44]. NF is based on the first-time mothers’ (and families) needs; hence, the intervention group may have received almost the same amount of follow-up as the control group. *Last*, our sample is biased toward higher sociodemographic status, which is associated with higher QoL in pregnant and postpartum women [7, 9]. Thus, we can assume that a possible ceiling effect toward a higher QoL was present in our sample and that the ability to detect an increase in QoL over time was limited.

Emotional and appraisal support were statistically significantly associated with higher QoL in the first-time mothers. This relationship has previously been sparsely explored due to predominant attention on general social support measurements [12, 13]. Clinically, measuring specific social support constructs seem essential to gain knowledge on sources and types of support needed to affect QoL domains in first-time mothers. Emotional and appraisal support could be considered a supporting presence and relates to being comforted, appreciated, cared for, and having someone to talk to and receive feedback from [14]. Emotional support impacted the QoL primarily in the social relationship domain, but also in the physical health domain. The number of formal and informal sources of support had no impact on this association. First-time mothers have identified their partner and mother as the primary emotional support source [20, 48]. Additionally, first-time mothers have stated that they want professionals to affirm their competence as a mother through emotional and appraisal support [22]. Altogether, this argues that PHNs and other healthcare professionals should strive to provide emotional support to first-time mothers and that this may positively affect their QoL domain levels.

Appraisal support was associated with the QoL domains environment and psychological. These domains encompass factors such as safety, home environment, self-esteem, feelings, and access to information and health care [37], and mental health components related to such factors have previously been found to be associated with social support [15–17]. The number of formal and informal sources did not impact the associations between appraisal support and the respective QoL domains. First-time mothers have identified PHNs, in addition to their partners and mothers, as a source of appraisal support [1, 20]. Thus, our results may imply that appraisal support provided by formal and informal sources facilitates first-time mothers in navigating the challenges and changes that come with becoming a mother, in line with previous studies [20, 21, 25]. Feedback and affirmation to first-time mothers attributed care for the baby and experiences of the postpartum period seem to increase the psychological and environmental QoL. Hence, PHNs and midwives should assess for and facilitate informal support and provide formal professional support during pregnancy and postpartum, supported by previous findings [22, 49]. In the context of the present study, NF enables the opportunity for PHNs to start this process already during pregnancy, a period traditionally reserved for midwives. However, the effect of emotional and appraisal support on QoL domain levels may be of limited clinical relevance as it is small. Further research in larger samples and through qualitative studies is needed to provide further insights into the relationships identified in our study.

Our findings suggest that informational and instrumental support may not play a crucial role in influencing the QoL of first-time mothers. Further, the findings may imply that emotional or appraisal support is more beneficial to improve QoL in first-time mothers. However, the results should be interpreted by Norway’s cultural and societal context to understand the clinical implications of this finding. Appropriate advice on infant care and behaviors with hands-on assistance may be readily available to our sample of first-time mothers from professionals and close ones. This may imply that the informational and instrumental support is sufficient enough. Further research utilizing different designs and measures is needed to better understand the role of informational and instrumental, as well as emotional and appraisal, support on the QoL of first-time mothers in Norway.

Our findings revealed that the QoL level in pregnancy was related to the level at three months postpartum. This association was found in all QoL domains, with its highest effect in the psychological and social relationship domains. In line with the intention of NF, this finding supports that to improve first-time mothers’ QoL levels, interventions should be started already during pregnancy. Extensive knowledge exists of factors associated with poor and good QoL that emerge during pregnancy [7] and postpartum [8]. To improve first-time mothers’ QoL, knowledge of these factors is essential for PHNs, midwives, and other healthcare professionals working with this population. Clinically, actions should be directed to inform PHNs and midwives on the importance of pregnant women’s health and how it could affect their health postpartum.

The perception of enough sleep at six weeks postpartum was associated with higher levels of QoL in all domains at three months postpartum, showing the highest impact in the physical health domain. A similar relationship was found in our sample during pregnancy [50], and challenges with sleep are previously found to affect the physical health QoL of postpartum women [51]. However, due to small and negligible effect sizes, the relationship between QoL domains and the perception of enough sleep may have limited to no clinical relevance. Nevertheless, it may imply that PHNs should be observant on the potential benefit of enough sleep on first-time mothers health-related QoL.

### Strengths and limitations

The strength of our study is the generation of new knowledge on Norwegian first-time mothers’ QoL and the program offered by the CHS related to QoL. In a Norwegian context, this population and the CHS are rarely subjects to research. Additionally, our study provides knowledge on the association between specific constructs of social support and QoL.

This study was not powered to reveal possible differences between the intervention and control group, thus we consider our findings to be exploratory. Our study has a recruitment rate slightly above 50% in both the intervention and control groups and a response rate of around 70–75% from T1 to T3. We acknowledge that the recruitment rate was low, however, it is not uncommon in studies of new parent populations. This limits the generalizability of our study. However, our results are valid for a sub-population of Norwegian women between 29–35 years, who are partnered or married, and have higher family income and educational levels.

Limitations and challenges of our study regarding the timeframe, fidelity and implementation, time points, response shift, the use of a generic QoL measure, complexity of the intervention, and biased sample, are already discussed above.

The measure of PICSS has not previously been validated in a Norwegian sample of postpartum women. For the New Families research project, the measure was translated and re-translated by professional translators, and we have provided Cronbach’s alpha for each dimension. Future studies should aim to provide further psychometric testing of the instrument in a Norwegian sample, including assurance of cultural adaptation.

## Conclusions

Our results did not reveal that NF has a statistically significant impact on the QoL of first-time mothers at three months postpartum. Our results suggest that NF does not harm the QoL of first-time mothers, and the intervention may be continued if found effective on other outcomes. Further research, including qualitative interviews, is needed to provide insights into the impact of NF. The association between the QoL level during pregnancy and postpartum suggests that postnatal interventions targeting improved QoL could potentially improve postpartum levels. Social support as emotional and appraisal support seems beneficial for first-time mothers’ QoL, indicating that PHNs may contribute by assessing and providing support and facilitating informal support to first-time mothers.

### Supplementary Information

**Additional file 1:** **Table S1.** Impact of New Families on WHOQOL-BREF domains at three months postpartum (T3) in first-time mothers who had answered before the outbreak of COVID-19 (*n* = 131).

**Additional file 2:** **Table S2.** Association between WHOQOL-BREF domains at T3, social support domains (PICSS) at T2 and selective possible predictive factors in first-time mothers who had answered before the outbreak of COVID-19 (*n* = 131).

## Data Availability

All relevant data are presented in this paper. Further records from this study are available from the corresponding author upon reasonable request.

## Abbreviations

QoL: Quality of Life
WHO: World Health Organization
CHS: Child Health Services
PHN: Public Health Nurse
WHOQOL-BREF: World Health Organization Quality of Life brief version
PICSS: Perinatal Infant Care Social Support scale
NF: New Families
