# Supplementary material for: The impact of New Families home visiting program on first-time mothers’ quality of life and its association with social support: a non-randomized controlled study
Source: BMC Public Health. 2023 Dec 8;23:2457. doi: 10.1186/s12889-023-17285-0 (PMC10704737; doi:10.1186/s12889-023-17285-0)
Supplement: Supplementary file 1 — Additional file 1: Table S1. Impact of New Families on WHOQOL-BREF domains at three months postpartum (T3) in first-time mothers who had answered before the outbreak of COVID-19 (n = 131). [file 12889_2023_17285_MOESM1_ESM.docx]

**Table S1**: Impact of New Families on WHOQOL-BREF domains at three months postpartum (T3) in first-time mothers who had answered before the outbreak of COVID-19 (n=131).

| T1 variables | QoL domain T3 | | | | | | | | | | | | | | | |
| --- | --- | --- | --- | --- | --- | --- | --- | --- | --- | --- | --- | --- | --- | --- | --- | --- |
|  | Physical health | | | | Psychological | | | | Social relationships | | | | Environmental | | | |
|  | B | 95% CI | ES | p-value | B | 95% CI | ES | p-value | B | 95% CI | ES | p-value | B | 95% CI | ES | p-value |
| Intervention (ref control) | -.52 | -4.20 to 3.16 | -.14 | .779 | 1.31 | -6.01 to 3.40 | -.27 | .583 | 3.71 | -1.06 to 8.49 | .67 | .126 | 2.41 | -3.19 to 8.02 | .63 | .396 |
| QoL domain | .29 | .09 to .48 | .39 | .004 | .70 | .46 to .94 | .64 | <.001 | .79 | .56 to 1.03 | .73 | <.001 | .66 | .40 to .92 | .61 | <.001 |
| Intervention*QoL domain | -.01 | -.25 to .24 | -.02 | .961 | .04 | .26 to .34 | .13 | .793 | -.23 | -.53 to .08 | -.64 | .146 | -.17 | -.49 to .16 | -.75 | .313 |
| Pregnancy week | .08 | -.01 to .17 | .16 | .077 | .05 | .05 to .14 | .07 | .324 | .06 | -.05 to .17 | .08 | .289 | .04 | -.04 to .12 | .08 | .327 |
| Family income (three levels) | .25 | -.17 to .68 | .10 | .242 | .34 | -.10 to .77 | .10 | .125 | -.27 | -.80 to .25 | -.07 | .306 | .14 | -.27 to .55 | .05 | .499 |
| Perception of sleep (ref not enough sleep) | .01 | -.64 to .66 | .00 | .980 | -.03 | -.68 to .62 | -.01 | .927 | .11 | -.68 to .90 | .02 | .778 | .09 | -.50 to .67 | .02 | .762 |
| *QoL = quality of life* | | | | | | | | | | | | | | | | |
