# Supplementary material for: The impact of New Families home visiting program on first-time mothers’ quality of life and its association with social support: a non-randomized controlled study
Source: BMC Public Health. 2023 Dec 8;23:2457. doi: 10.1186/s12889-023-17285-0 (PMC10704737; doi:10.1186/s12889-023-17285-0)
Supplement: Supplementary file 2 — Additional file 2: Table S2. Association between WHOQOL-BREF domains at T3, social support domains (PICSS) at T2 and selective possible predictive factors in first-time mothers who had answered before the outbreak of COVID-19 (n = 131). [file 12889_2023_17285_MOESM2_ESM.docx]

Table S2: Association between WHOQOL-BREF domains at T3, social support domains (PICSS) at T2 and selective possible predictive factors in first-time mothers who had answered before the outbreak of COVID-19 (n=131)

|  | **Step One**  QoL domains and social support domains entered | | | | | | | | **Step two**  Statistically significant from step one and possible selective predictive factors added | | | | | | | |
| --- | --- | --- | --- | --- | --- | --- | --- | --- | --- | --- | --- | --- | --- | --- | --- | --- |
|  | **Full model** | | | | **Final model** | | | | **Full model** | | | | **Final model** | | | |
|  | **B** | **95% CI** | **ES** | **p-value** | **B** | **95% CI** | **ES** | **p-value** | **B** | **95% CI** | **ES** | **p-value** | **B** | **95% CI** | **ES** | **p-value** |
| ***Physical health domain*** | | | | | | | | | | | | | | | | |
| PICSS Informational | .11 | .00 to .23 | .19 | .050 | .12 | .01 to .23 | .20 | .036 | .09 | -.01 to .18 | .15 | .078 | **.10** | **.01 to .18** | **.16** | **.030** |
| PICSS Instrumental | -.10 | -.20 to .01 | -.17 | .073 | -.09 | -.19 to .02 | -.16 | .095 |  |  |  |  |  |  |  |  |
| PICSS Emotional | .18 | -.18 to .55 | .15 | .324 | .29 | .07 to .50 | .23 | .009 | .05 | -.15 to .26 | .04 | .616 |  |  |  |  |
| PICSS Appraisal | .13 | -.23 to .49 | .11 | .471 |  |  |  |  |  |  |  |  |  |  |  |  |
| PICSS Informal support | .12 | -.07 to .30 | .10 | .208 |  |  |  |  |  |  |  |  |  |  |  |  |
| PICSS Formal support | -.21 | -.49 to .07 | -.12 | .139 |  |  |  |  |  |  |  |  |  |  |  |  |
| Physical health QoL domain, T1 | .29 | .17 to .41 | .37 | <.001 | .30 | .18 to .42 | .38 | <.001 | .27 | .16 to .39 | .36 | <.001 | **.25** | **.14 to .36** | **.34** | **<.001** |
| Pregnancy week T1 |  |  |  |  |  |  |  |  | .04 | -.04 to .12 | .08 | .306 |  |  |  |  |
| Age of mother |  |  |  |  |  |  |  |  | -.08 | -.15 to -.01 | -.16 | .036 | -.07 | -.14 to .00 | -.15 | .054 |
| Perception of sleep, T1 (ref not enough sleep) |  |  |  |  |  |  |  |  | -.30 | -.90 to .31 | -.08 | .334 |  |  |  |  |
| Perception of sleep, T2 (ref not enough sleep) |  |  |  |  |  |  |  |  | 1.41 | .83 to 1.99 | .38 | <.001 | **1.43** | **.88 to 1.97** | **.38** | **<.001** |
| Family income (three levels) |  |  |  |  |  |  |  |  | .34 | -.06 to .73 | .13 | .094 | .34 | -.05 to .72 | .13 | .088 |
| ***Psychological domain*** | | | | | | | | | | | | | | | | |
| PICSS Informational | .06 | -.05 to .17 | .08 | .308 |  |  |  |  |  |  |  |  |  |  |  |  |
| PICSS Instrumental | -.09 | -.19 to .01 | -.13 | .076 |  |  |  |  |  |  |  |  |  |  |  |  |
| PICSS Emotional | -.04 | -.38 to .31 | -.02 | .845 |  |  |  |  |  |  |  |  |  |  |  |  |
| PICSS Appraisal | .48 | .14 to .83 | .33 | .007 | .42 | .24 to .60 | .29 | <.001 | .33 | .16 to .50 | .25 | <.001 | **.34** | **.17 to .51** | **.25** | **<.001** |
| PICSS Informal support | -.04 | -.21 to .14 | -.03 | 678 |  |  |  |  |  |  |  |  |  |  |  |  |
| PICSS Formal support | .02 | -.24 to .29 | .01 | .861 |  |  |  |  |  |  |  |  |  |  |  |  |
| Psychological QoL domain, T1 | .63 | .50 to .76 | .60 | <.001 | .64 | .51 to .77 | .61 | <.001 | .64 | .50 to .78 | .59 | <.001 | **.64** | **.50 to .77** | **.59** | **<.001** |
| Pregnancy week T1 |  |  |  |  |  |  |  |  | .02 | -.06 to .10 | .03 | .613 |  |  |  |  |
| Age of mother |  |  |  |  |  |  |  |  | .01 | -.06 to .09 | .02 | .696 |  |  |  |  |
| Perception of sleep, T1 (ref not enough sleep) |  |  |  |  |  |  |  |  | -.01 | -.61 to .59 | -.00 | .969 |  |  |  |  |
| Perception of sleep, T2 (ref not enough sleep) |  |  |  |  |  |  |  |  | .58 | -.02 to 1.17 | .13 | .057 | .57 | -.01 to 1.15 | .12 | .054 |
| Family income (three levels) |  |  |  |  |  |  |  |  | .29 | -.11 to .69 | .09 | .147 |  |  |  |  |
| ***Social relationships*** | | | | | | | | | | | | | | | | |
| PICSS Informational | .05 | -.09 to .19 | .06 | .504 |  |  |  |  |  |  |  |  |  |  |  |  |
| PICSS Instrumental | -.08 | -.21 to .05 | -.10 | .224 |  |  |  |  |  |  |  |  |  |  |  |  |
| PICSS Emotional | .19 | -.26 to .65 | .11 | .406 |  |  |  |  |  |  |  |  |  |  |  |  |
| PICSS Appraisal | .31 | -.14 to .75 | .18 | .175 | .42 | .18 to .66 | .25 | .001 | .34 | .13 to .55 | .22 | .002 | **.33** | **.12 to .54** | **.22** | **.002** |
| PICSS Informal support | .18 | -.04 to .40 | .11 | .110 |  |  |  |  |  |  |  |  |  |  |  |  |
| PICSS Formal support | -.19 | -.53 to 0.16 | -.07 | .288 |  |  |  |  |  |  |  |  |  |  |  |  |
| Social relationship QoL domain, T1 | .56 | .40 to .72 | .51 | <.001 | .58 | .43 to .74 | .53 | <.001 | .52 | .37 to .67 | .49 | <.001 | **.52** | **.38 to .67** | **.49** | **<.001** |
| Pregnancy week T1 |  |  |  |  |  |  |  |  | .07 | -.03 to .17 | .09 | .155 |  |  |  |  |
| Age of mother |  |  |  |  |  |  |  |  | -.09 | -.18 to-.00 | -.14 | .044 | -.09 | -.17 to .00 | -.13 | .050 |
| Perception of sleep, T1 (ref not enough sleep) |  |  |  |  |  |  |  |  | -.12 | -.86 to .62 | -.02 | .748 |  |  |  |  |
| Perception of sleep, T2 (ref not enough sleep) |  |  |  |  |  |  |  |  | .95 | .22 to 1.68 | .18 | .012 | **.98** | **.26 to 1.69** | **.18** | **.008** |
| Family income (three levels) |  |  |  |  |  |  |  |  | -.06 | -.55 to .43 | -.02 | .803 |  |  |  |  |
| ***Environment*** | | | | | | | | | | | | | | | | |
| PICSS Informational | .09 | -.01 to .18 | .15 | .083 |  |  |  |  |  |  |  |  |  |  |  |  |
| PICSS Instrumental | -.05 | -.14 to .04 | -.09 | .255 |  |  |  |  |  |  |  |  |  |  |  |  |
| PICSS Emotional | -.15 | -.46 to .16 | -.13 | .338 |  |  |  |  |  |  |  |  |  |  |  |  |
| PICSS Appraisal | .50 | .19 to .81 | .43 | .002 | .40 | .24 to .56 | .35 | <.001 | .34 | .18 to .50 | .32 | <.001 | **.34** | **.19 to .50** | **.32** | **<.001** |
| PICSS Informal support | .03 | -.13 to .18 | .03 | .718 |  |  |  |  |  |  |  |  |  |  |  |  |
| PICSS Formal support | .02 | -.22 to .26 | .01 | .871 |  |  |  |  |  |  |  |  |  |  |  |  |
| Environment domain QoL, T1 | .44 | .29 to .59 | .43 | <.001 | .45 | .31 to .60 | .44 | <.001 | .39 | .23 to .56 | .37 | <.001 | **.39** | **.23 to .55** | **.37** | **<.001** |
| Pregnancy week T1 |  |  |  |  |  |  |  |  | .02 | -.05 to .09 | .04 | .592 |  |  |  |  |
| Age of mother |  |  |  |  |  |  |  |  | -.06 | -.13 to .00 | -.14 | .050 | **-.06** | **-.13 to .00** | **-.14** | **.049** |
| Perception of sleep, T1 (ref not enough sleep) |  |  |  |  |  |  |  |  | .01 | -.53 to .55 | .00 | .962 |  |  |  |  |
| Perception of sleep, T2 (ref not enough sleep) |  |  |  |  |  |  |  |  | .54 | .02 to 1.07 | .15 | .044 | **.56** | **.05 to 1.07** | **.15** | **.032** |
| Family income (three levels) |  |  |  |  |  |  |  |  | .30 | -.08 to .68 | .12 | .115 | .32 | -.06 to .69 | .12 | .095 |
| *QoL = quality of life* | | | | | | | | | | | | | | | | |
